# Supplementary material for: Identification of Novel Insulin Resistance Related ceRNA Network in T2DM and Its Potential Editing by CRISPR/Cas9
Source: Int J Mol Sci. 2021 Jul 29;22(15):8129. doi: 10.3390/ijms22158129 (PMC8348752; doi:10.3390/ijms22158129)
Supplement: Supplementary file 1 [file ijms-22-08129-s001.zip › ijms-1287053-supplementary.pdf]

figure S1: ROC curve analysis for differential expression of investigated network panel between T2DM and control

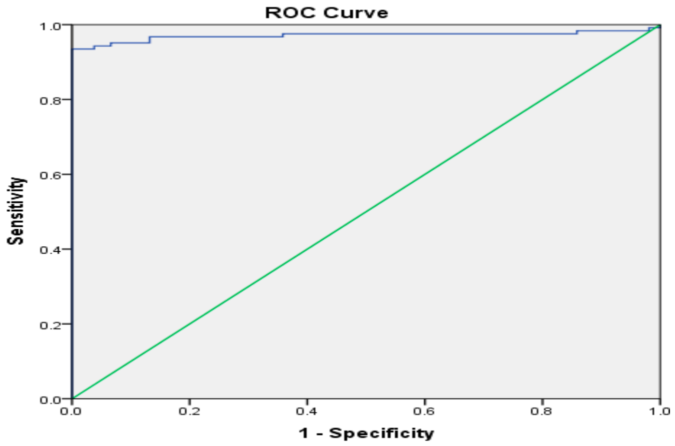

ROC curve of *RP11-773H22.4 lncRNA* (AUC: 0.971,95% CI: 0.944-0.998, P<0.01).

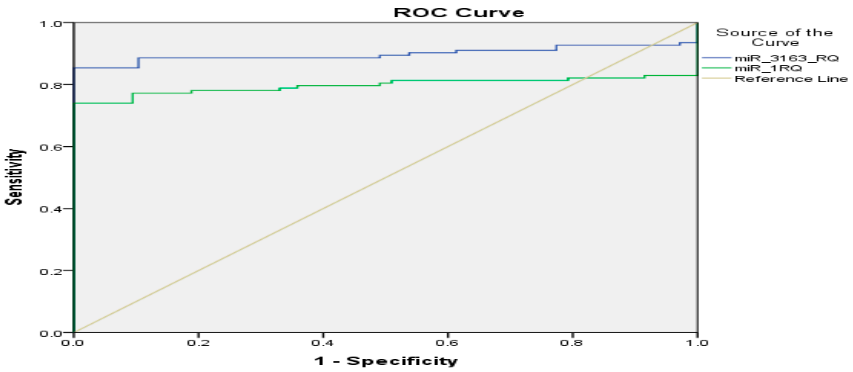

ROC curve of *miR-3163 miRNA (*AUC: 0.898, 95% CI: 0.848-0.948, P<0.01). *and mir-1 (*AUC: 0.797, 95% CI: 0.729-0.865, P<0.01)

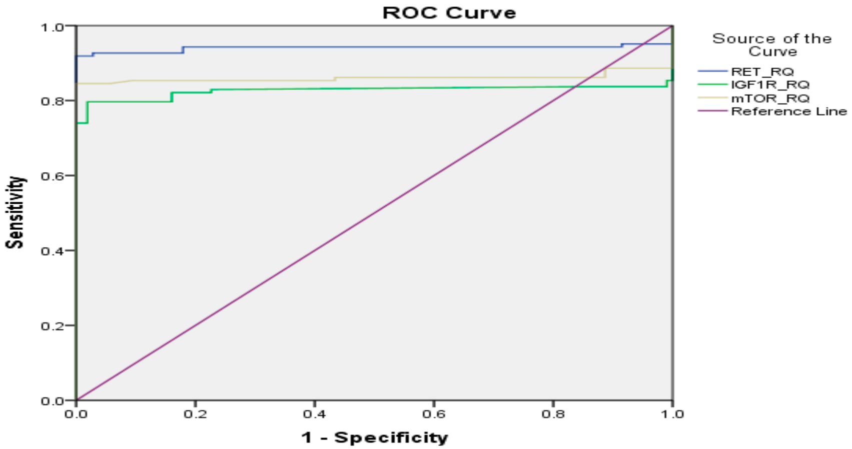

ROC curve for *RET mRNA* (AUC:0.941, 95% CI: 0.900-0.981, P<0.01)*, IGF1-R (*AUC: 0.826,95% CI: 0.761-0.892, P<0.01) *and m-TOR (*AUC : 0.860, 95% CI: 0.800-0.920, P<0.01).

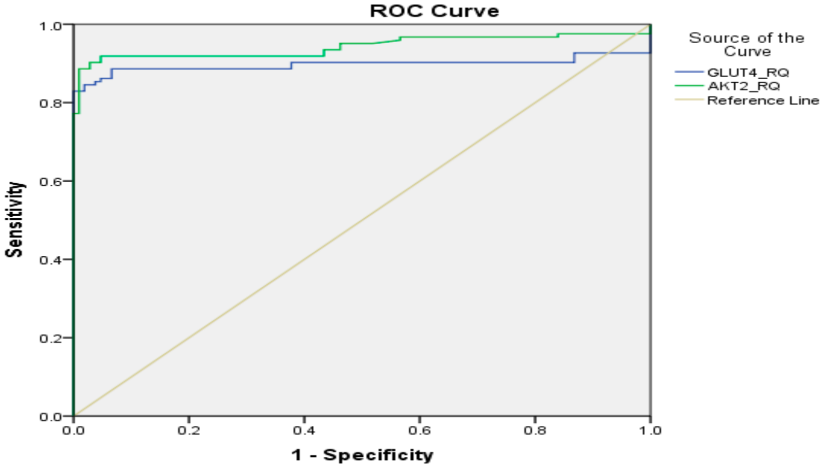

ROC curve for *GLUT4 mRNA (*AUC: 0.897, (95% CI: 0.846-0.948, P<0.01)*, AKT-2 (*AUC: 0.943, 95% CI: 0.908-0.978, P<0.01).

Table S1: Comparison between lymphocytes' count and viability among the 3 investigated cell line groups:

Dependent Variable	(I) Cell Lines	(J) Cell Lines	Mean Difference (I-J)	Std. Error	Sig.	95% Confidence Interval	
						Lower Bound	Upper Bound
Lymphocytes Count (x10*4)	T2DM lymphocytes without Cas9 editing	T2DM lymphocytes with Cas9 editing	5.000	2.735	NS .117	-1.69	11.69
		Healthy Control lymphocytes	-6.667	2.735	.051	-13.36	.03
	T2DM lymphocytes with Cas9 editing	T2DM lymphocytes without Cas9editing	-5.000	2.735	NS .117	-11.69	1.69
		Healthy Control lymphocytes	-11.667*	2.735	.005**	-18.36	-4.97

	Healthy Control lymphocytes	T2DM lymphocytes without Cas9 editing	6.667	2.735	.051	-.03	13.36
		T2DM lymphocytes with Cas9 editing	11.667*	2.735	.005**	4.97	18.36
**Lymphocytes Viability (%)**	T2DM lymphocytes without Cas9 editing	T2DM lymphocytes with Cas9 editing	1.5000	.6242	.053	-.027	3.027
		Healthy Control lymphocytes	-1.6333*	.6242	.040*	-3.161	-.106
	T2DM lymphocytes with Cas9 editing	T2DM lymphocytes without Cas9 editing	-1.5000	.6242	.053	-3.027	.027
		Healthy Control lymphocytes	-3.1333*	.6242	.002**	-4.661	-1.606
	Healthy Control lymphocytes	T2DM lymphocytes without Cas9 editing	1.6333*	.6242	.040*	.106	3.161
		T2DM lymphocytes with Cas9 editing	3.1333*	.6242	.002**	1.606	4.661

One-way ANOVA, Post Hoc test, p: NS, not significant (>0.05), **p < 0.01: is highly significant, *p<0.05: is significant.

figure S2(a): Expression of *RET mRNA* in insulin resistance and T2DM in Gene Atlas Database. Snapshot shows the expression of *RET* gene in T2DM ***(available at https://www.ebi.ac.uk)*** accessed on 9th of June 2017.

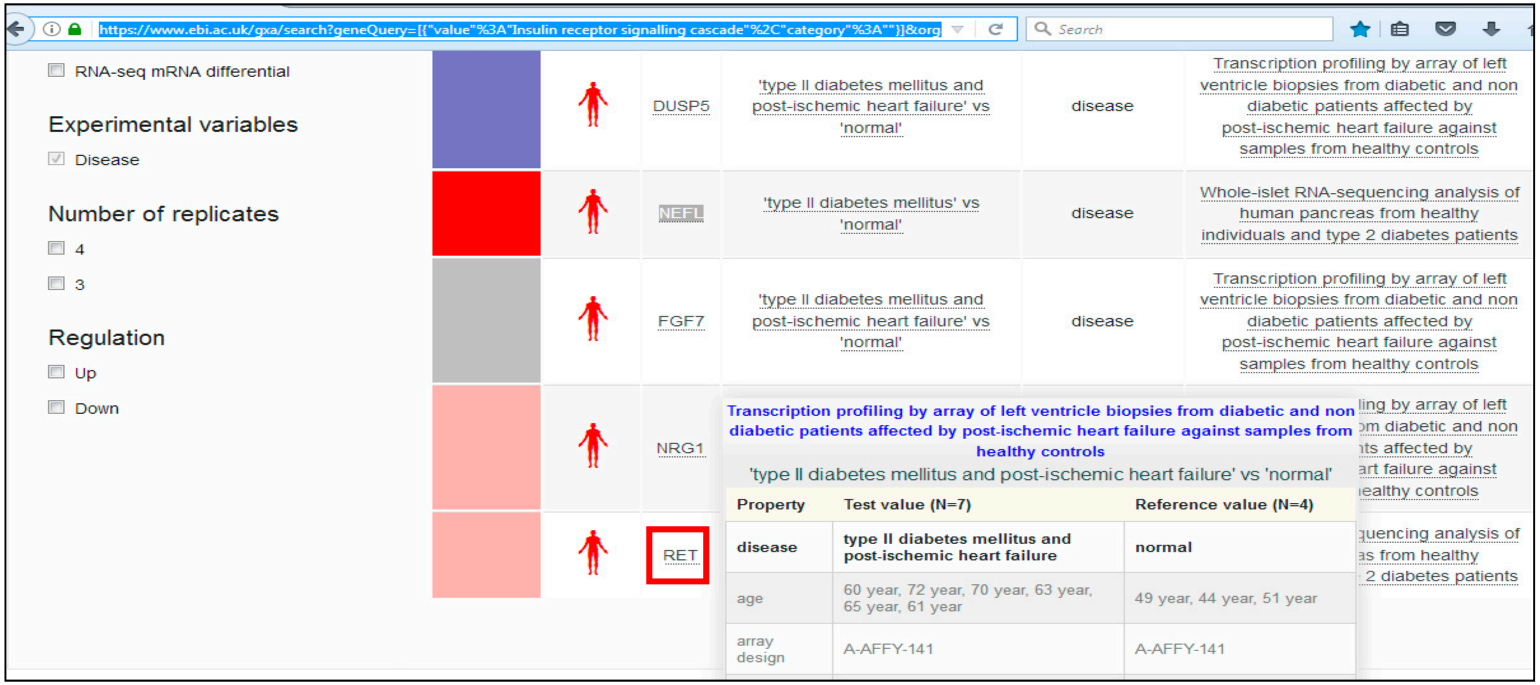

figure S2(b): Differential expression of *RET mRNA* in normal adipose and skeletal muscle in Protein Atlas Database . Snapshot shows the expression of *RET mRNA* in normal skeletal muscle and adipose tissue ***(available at https://www.proteinatlas.org/)*** accessed on 6th of September 2017.

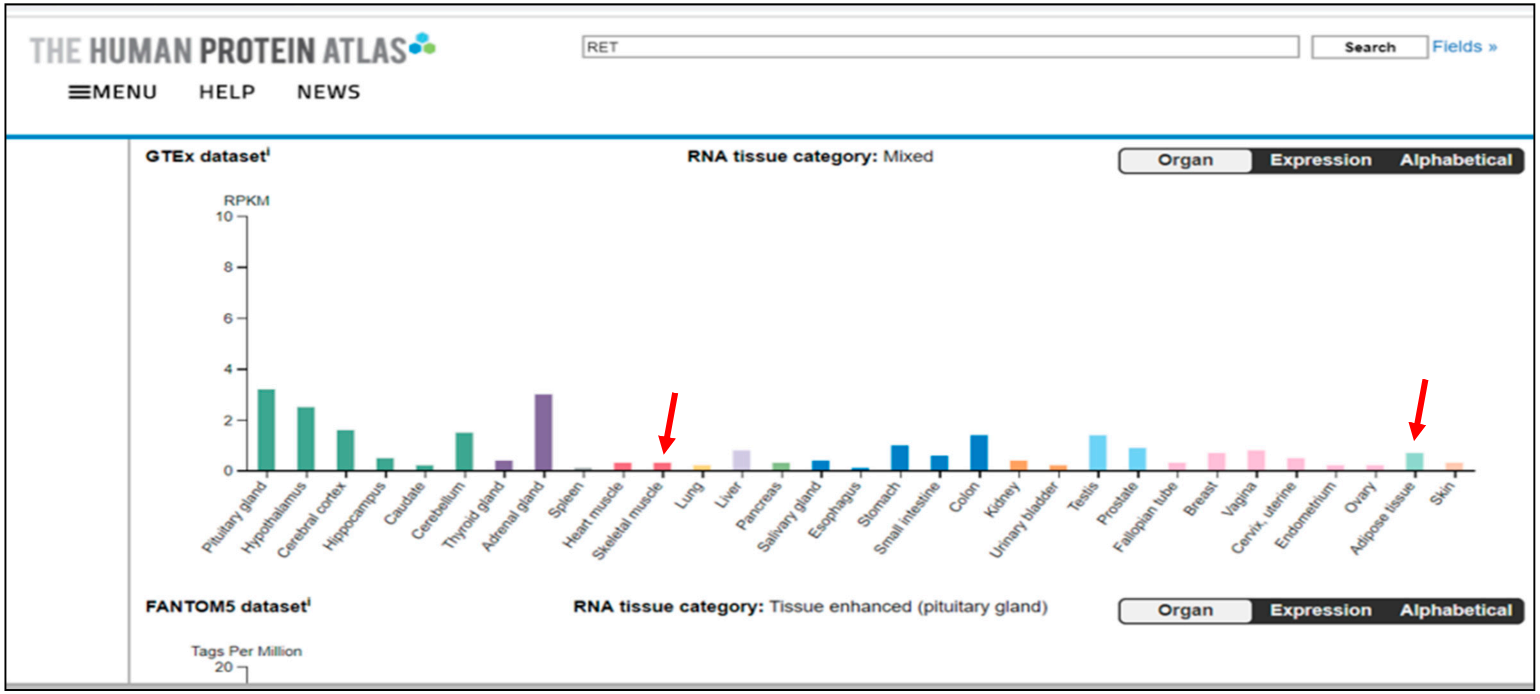

figure S2(c): Verification of expression of *RET mRNA* in normal skeletal muscle, adipose tissue and in WBCs. Snapshot shows the verification of expression of *RET mRNA* in normal skeletal muscle and adipose tissue and in WBCs ***(available at https://www.genecards.org/)***

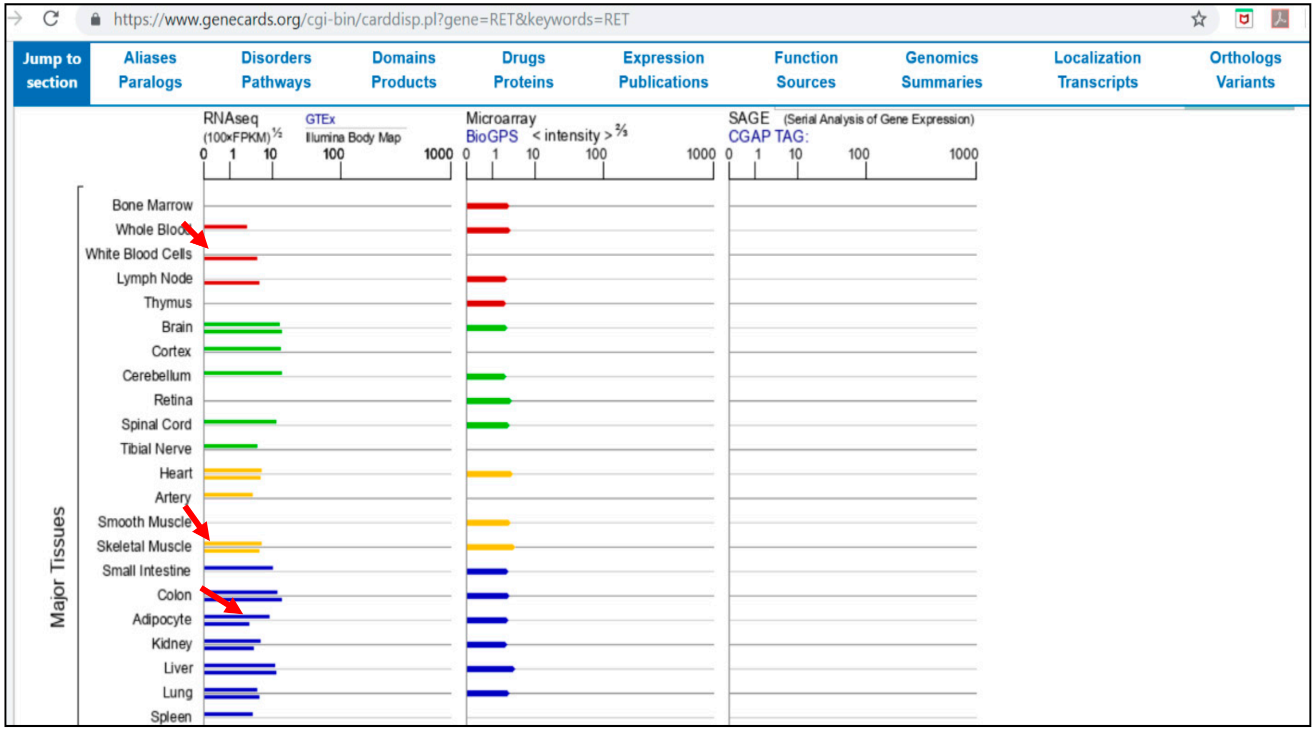

figure S2(d): Expression of *mTOR mRNA* in insulin resistance and T2DM in Gene Atlas Database (figure 4s). Snapshot shows the expression of *mTOR* gene in IR ***(available at https://www.ebi.ac.uk)***

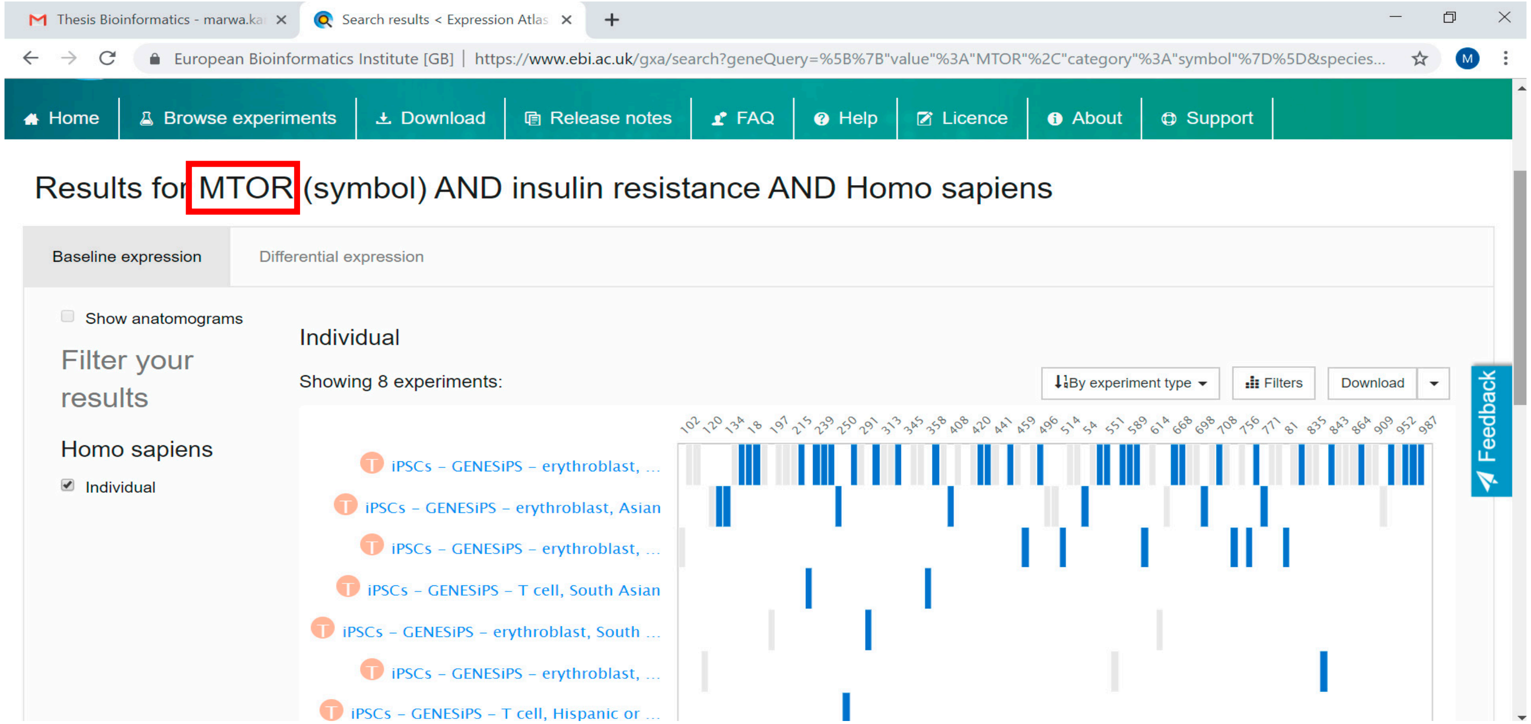

figure S2(e)**:** k map and string map show involvement of *mTOR* gene in insulin signaling pathway and its relation to *RET* gene in insulin resistance in T2DM. Snapshot verifying that the identified *mTOR* gene is linked to IR in T2DM ***(available at http://www.kegg.jp/)***

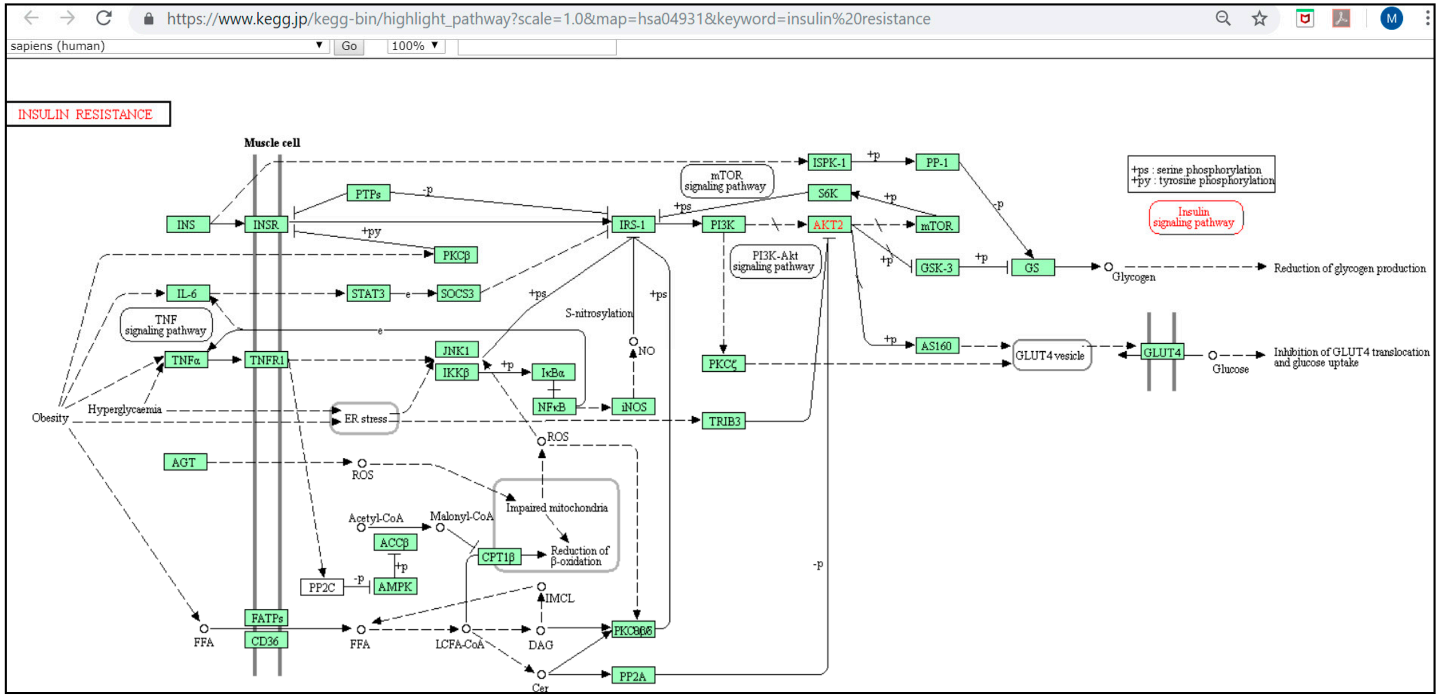

figure S2(f)**:** Snapshot shows verification of the identified candidate genes interaction in the pathogenesis of IR in T2DM (available at: http://string-db.org/

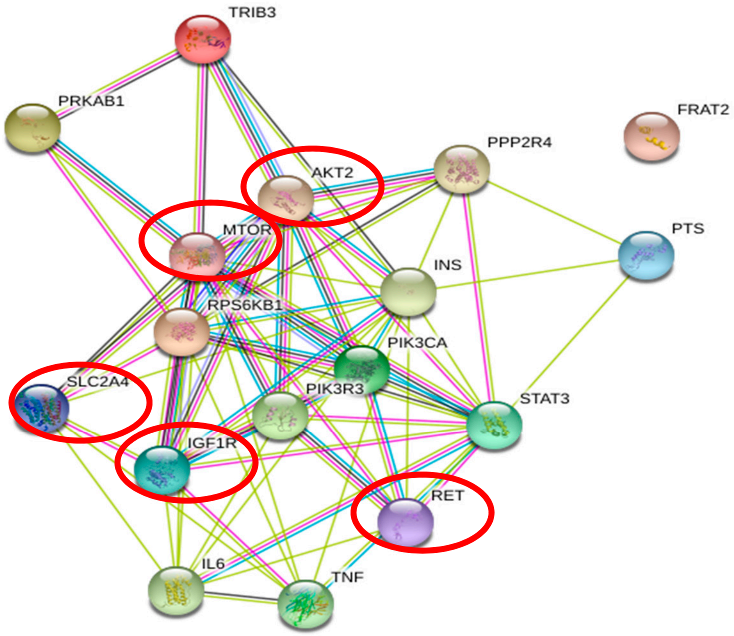

figure S2(g): Retrieval of long non-coding RNA *LncRNA-RP11-773H22.4* and the associated miRNAs: *miR-3163 & miR-1* that acting as ceRNAs for *RET mRNA* through Database of LncRNA acting as competing endogenous. Snapshot shows search for *LncRNA-RP11-773H22.4*(ENSG00000266969) and *miR-3163 miRNA* (available at http://gyanxet-beta.com/lncedb/index.php) accessed on 18th of July 2017.

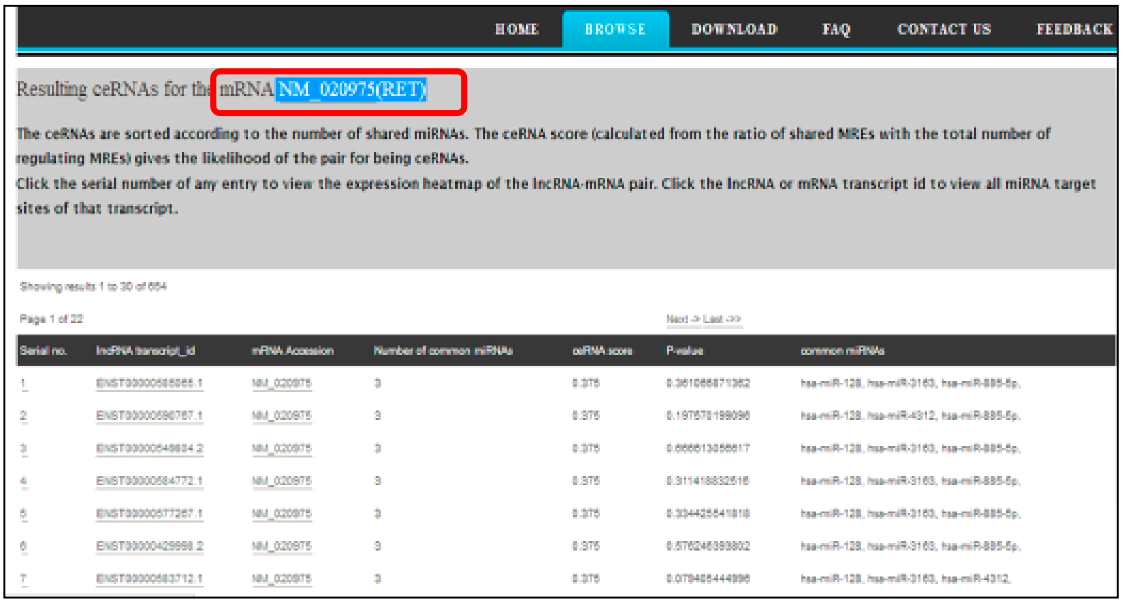

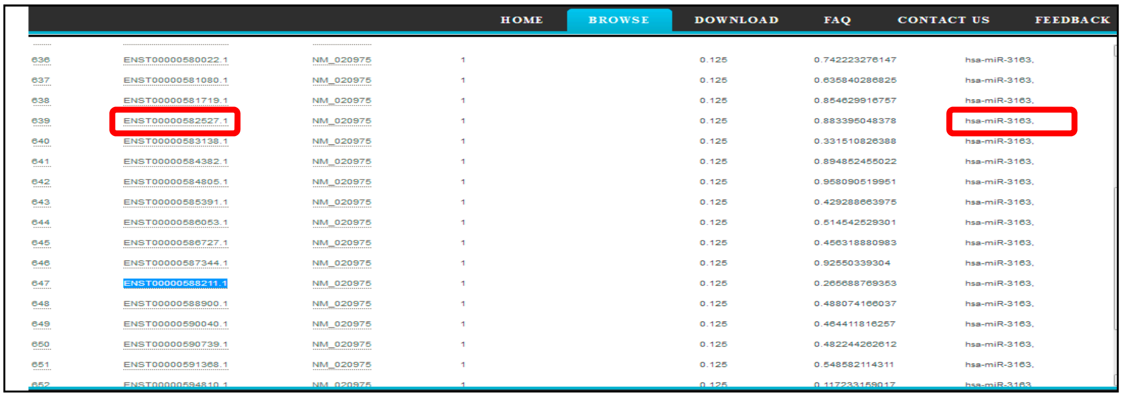

figure S2(h)**:** Snapshot shows identification of *miR-1 miRNA* related to *LncRNA-RP11-773H22.4* in T2DM (available at http://gyanxet-beta.com/lncedb/index.php) accessed on 18th of July 2017.

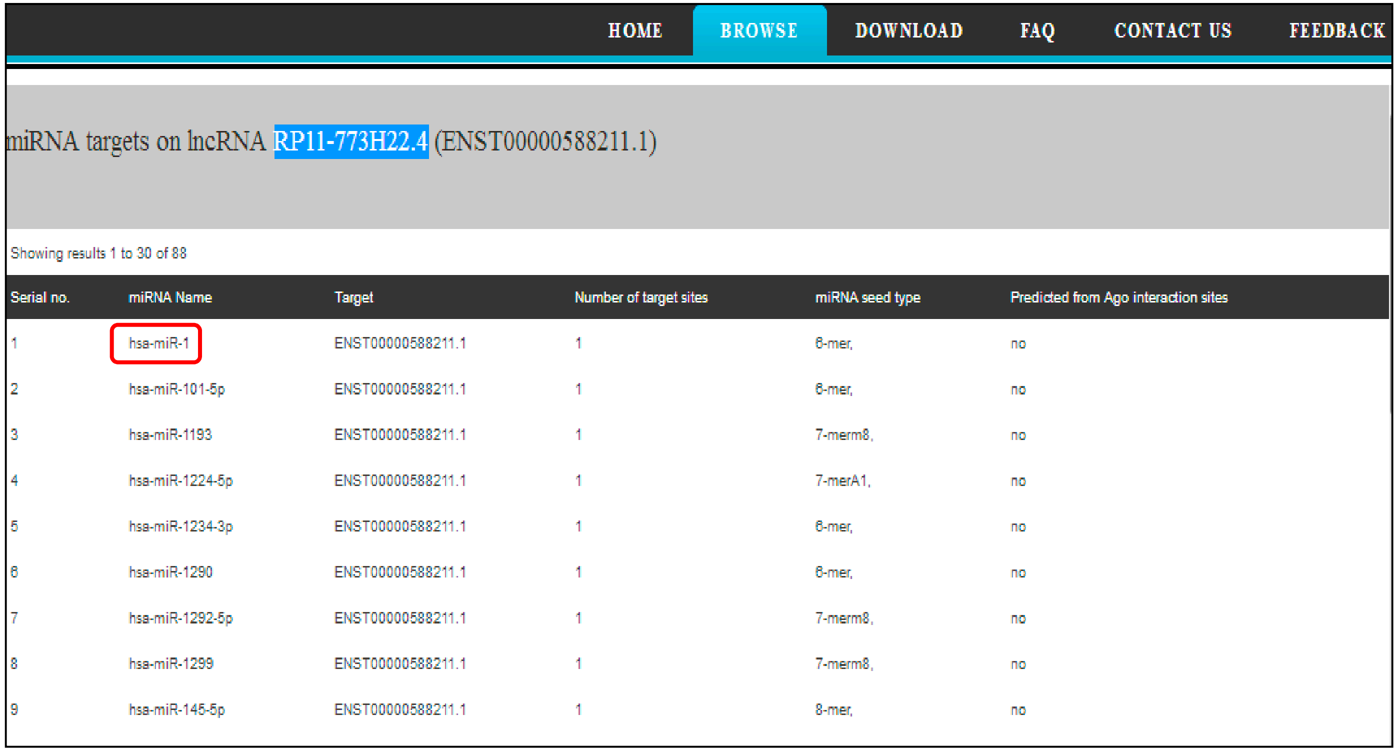

HOME BROWSE DOWNLOAD FAQ CONTACT US FEEDBACK

miRNA targets on lncRNA RP11-773H22.4 (ENST00000588211.1)

Showing results 1 to 30 of 88

Serial no.	miRNA Name	Target	Number of target sites	miRNA seed type	Predicted from Ago interaction sites
1	hsa-miR-1	ENST00000588211.1	1	6-mer,	no
2	hsa-miR-101-5p	ENST00000588211.1	1	6-mer,	no
3	hsa-miR-1193	ENST00000588211.1	1	7-merm8,	no
4	hsa-miR-1224-5p	ENST00000588211.1	1	7-merA1,	no
5	hsa-miR-1234-3p	ENST00000588211.1	1	6-mer,	no
6	hsa-miR-1290	ENST00000588211.1	1	6-mer,	no
7	hsa-miR-1292-5p	ENST00000588211.1	1	7-merm8,	no
8	hsa-miR-1299	ENST00000588211.1	1	7-merm8,	no
9	hsa-miR-145-5p	ENST00000588211.1	1	8-mer,	no

figure S2(i): Verification of *miR-3136* and *miR-1 miRNAs* that act as epigenetic regulators for RET and *mTOR mRNAs* in IR in T2DM. Snapshot shows identification of *m-TOR mRNA* related to *miR-3163miRNA* in IR and in T2DM ***(available at http://mirwalk.umm.uni-heidelberg.de/)*** accessed on 23rd of December 2017.

miRWalk

HOME FAQ RESOURCES ABOUT

hsa-miR-3163

Mirnaid hsa-miR-3163
Mimatid MIMAT0015037
SequenceUAUAAAAUGAGGGCAGUAAGAC

Interactions:

Mirna	Ensemblid	Genesymbol	Score	Position	Binding Site	Au	Me	N Pairings	Targetscan	Mirdb	Mirtarbase
hsa-miR-3163	ENST00000270776	PGD	0.92	CDS	1390,1408	0.47	-19.424	14	—	—	—
hsa-miR-3163	ENST00000377036	DFFA	0.92	3UTR	2141,2154	0.53	-16.49	12	—	—	—
hsa-miR-3163	ENST00000377022	CASZ1	0.92	CDS	2963,3003	0.34	-17.11	10	—	—	—
hsa-miR-3163	ENST00000344008	CASZ1	0.92	CDS	2963,3003	0.34	-17.11	10	—	—	—
hsa-miR-3163	ENST00000376936	EXOSC10	1.00	CDS	2136,2174	0.48	-17.11	13	—	—	—
hsa-miR-3163	ENST00000361445	MTOR	1.00	CDS	1664,1674	0.37	-17.11	9	—	—	—
hsa-miR-3163	ENST00000361445	MTOR	0.92	CDS	1331,1344	0.47	-17.11	12	—	—	—
hsa-miR-3163	ENST00000294484	DISP3	0.92	CDS	1555,1569	0.43	-16.623	12	—	—	—
hsa-miR-3163	ENST00000376770	FBXO44	1.00	5UTR	323,347	0.48	-6.515	13	—	—	—
hsa-miR-3163	ENST00000376768	FBXO44	0.92	3UTR	1457,1474	0.35	-17.11	10	—	—	—
hsa-miR-3163	ENST00000376590	MTHFR	0.85	3UTR	2922,2935	0.43	-14.908	12	—	—	—
hsa-miR-3163	ENST00000376590	MTHFR	0.85	CDS	616,628	0.35	-19.935	10	—	—	—

figure S2(j)**:** Verification of expression of *LncRNA-RP11-773H22.4* in normal skeletal muscle, adipose tissue and in WBCs. Snapshot shows the verification of expression of *LncRNA-RP11-773H22.4* or ENSG00000266969 in normal skeletal muscle, adipose tissue and in WBCs (available at http://www.noncode.org/)

Isoforms

Transcript ID

Data Sources

Source Name	Old Id
PMID26117828	PNCT_HSA052403
NAME	RP11-773H22.4
ensembl	ENST00000588211
PMID26117828	TCONS_00018126
gencode	ENST00000588211.1
NONCODEv4	NONHSAT058407
Incipedia	Inc-PTPN2-6-1_dup1

figure S2(k): Verification of expression of *miR-3163 miRNA* in normal skeletal muscle and adipose tissue.

www.noncode.org/show_rna.php?id=NONHSAT058407&version=2&utd=1#

Search

Expression Profile(Data Source:Human Body Map)

adipose	adrenal	brain	brain_R	breast	colon	foreskin	heart
7.60155e-09	0.652	0.538983	0.254605	1.22386	1.94835e-09	2.15761e-09	0.259929
**hela_R**	**HLF_1**	**HLF_2**	**kidney**	**liver**	**liver_R**	**lung**	**lymphNode**
0.262999	0.700997	1.60823e-06	0.3017	0.713647	1.36268e-12	5.15849e-09	0.787238
**ovary**	**placenta_R**	**prostate**	**skeltalMuscle**	**testes**	**testes_R**	**thyroid**	**whiteBloodCell**
0.973065	4.95728e-08	0.599253	1.79893e-35	0.369595	1.05324	0.75897	6.92361e-08

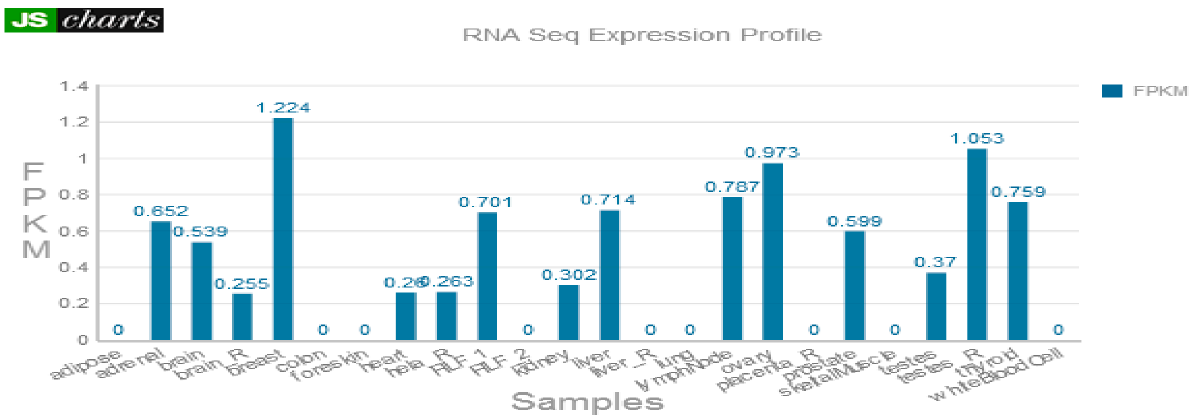

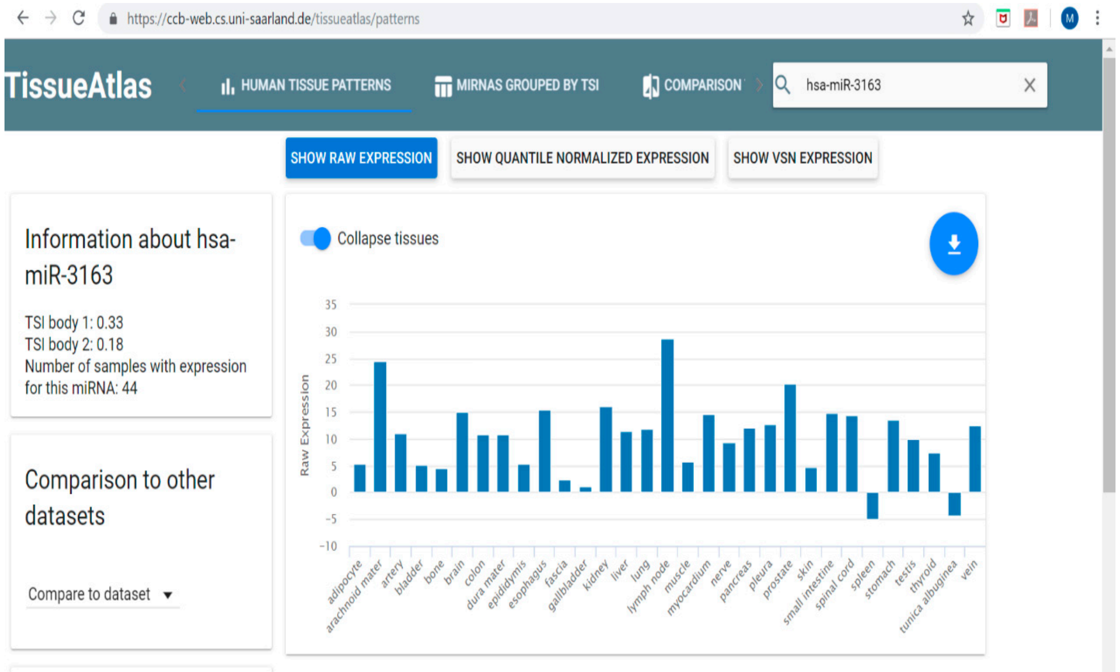
https://ccb-web.cs.uni-saarland.de/tissueatlas/patterns
TissueAtlas
HUMAN TISSUE PATTERNS
MIRNAS GROUPED BY TSI
COMPARISON
hsa-miR-3163
SHOW RAW EXPRESSION
SHOW QUANTILE NORMALIZED EXPRESSION
SHOW VSN EXPRESSION
Information about hsa-miR-3163
TSI body 1: 0.33
TSI body 2: 0.18
Number of samples with expression for this miRNA: 44
Comparison to other datasets
Compare to dataset
Collapse tissues
Raw Expression
35
30
25
20
15
10
5
0
-5
-10
adipocyte
arachnoid mater
artery
bladder
bone
brain
colon
dura mater
epididymis
esophagus
fascia
gallbladder
kidney
liver
lung
lymph node
muscle
myocardium
nerve
pancreas
pleura
prostate
skin
small intestine
spinal cord
spleen
stomach
testis
thyroid
tunica albuginea
vein

figure S2(l): Alignment between *LncRNA-RP11-773H22.4* and *miR-3163 miRNA* (***available at https://www.ebi.ac.uk)***

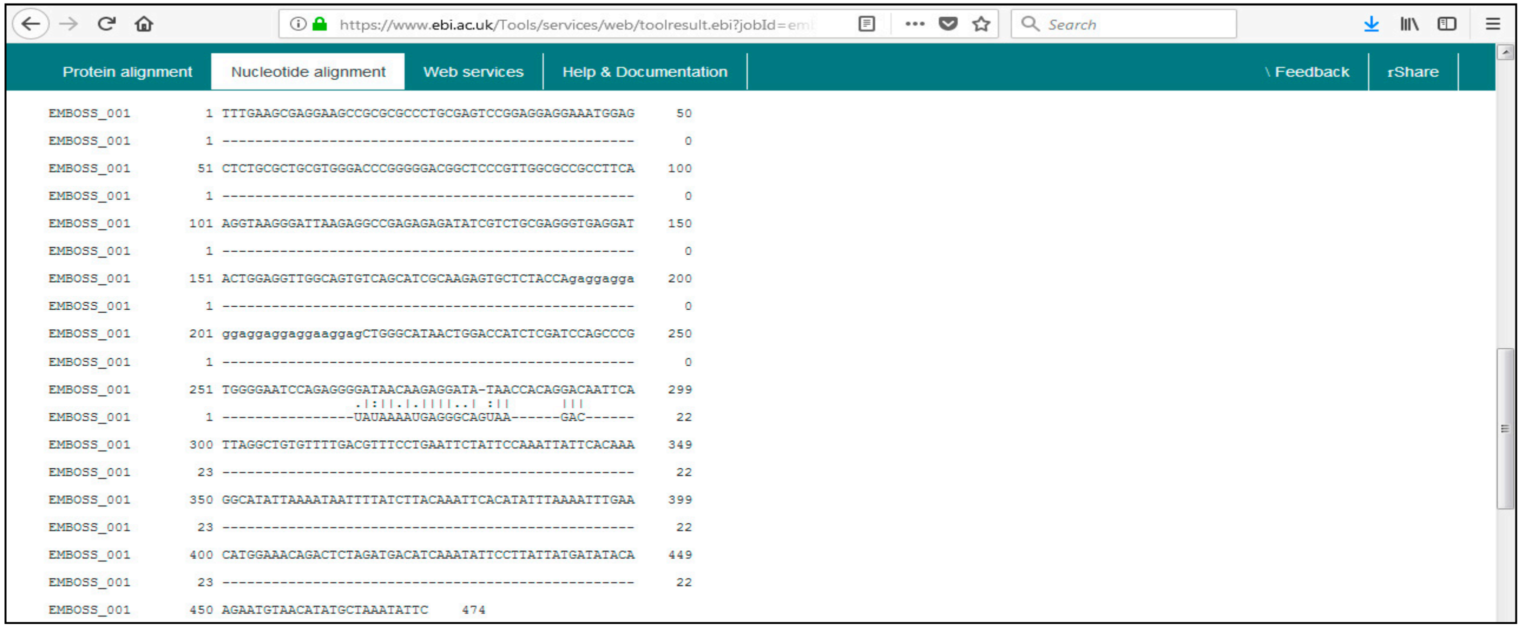


```
EMBOSS_001      1 TTTGAAGCGAGGAAGCCGCGCCCTGCGAGTCCGGAGGAGGAAATGGAG     50
EMBOSS_001      1 --------------------------------------------------      0
EMBOSS_001     51 CTCTGCGCTGCGTGGGACCCGGGGGACGGCTCCCGTTGGCGCCGCCTTCA    100
EMBOSS_001      1 --------------------------------------------------      0
EMBOSS_001    101 AGGTAAGGGATTAAGAGGCCGAGAGAGATATCGTCTGCGAGGGTGAGGAT    150
EMBOSS_001      1 --------------------------------------------------      0
EMBOSS_001    151 ACTGGAGGTTGGCAGTGTCAGCATCGCAAGAGTGCTCTACCAgaggagga    200
EMBOSS_001      1 --------------------------------------------------      0
EMBOSS_001    201 ggaggaggaggaaggagCTGGGCATAACTGGACCATCTCGATCCAGCCCG    250
EMBOSS_001      1 --------------------------------------------------      0
EMBOSS_001    251 TGGGGAATCCAGAGGGGATAACAAGAGGATA-TAACCACAGGACAATTCA    299
                                  .|:||.|.||||..| :||      |||
EMBOSS_001      1 ----------------UAUAAAAUGAGGGCAGUAA------GAC------     22
EMBOSS_001    300 TTAGGCTGTGTTTTGACGTTTCCTGAATTCTATTCCAAATTATTCACAAA    349
EMBOSS_001     23 --------------------------------------------------     22
EMBOSS_001    350 GGCATATTAAAATAATTTTATCTTACAAATTCACATATTTAAAATTTGAA    399
EMBOSS_001     23 --------------------------------------------------     22
EMBOSS_001    400 CATGGAAACAGACTCTAGATGACATCAAATATTCCTTATTATGATATACA    449
EMBOSS_001     23 --------------------------------------------------     22
EMBOSS_001    450 AGAATGTAACATATGCTAAATATTC    474
```

figure S2(m): Snapshot shows alignment of *miR-1* *miRNA* with *LncRNA-RP11-773H22.4* ***(available at https://www.ebi.ac.uk)*** accessed on 11th April 2019.

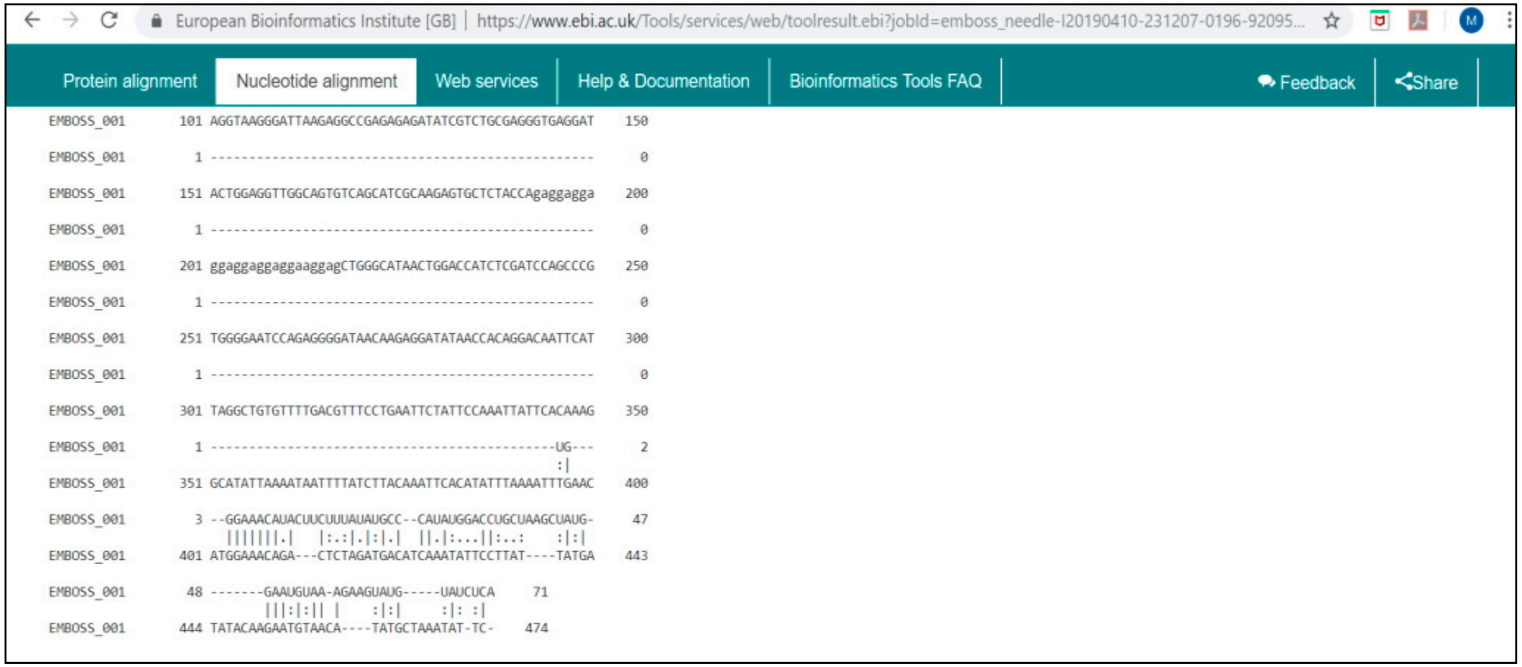

figure S2(n): Alignment between *mTOR mRNA miR-3163* ***(available at https://www.ebi.ac.uk)***

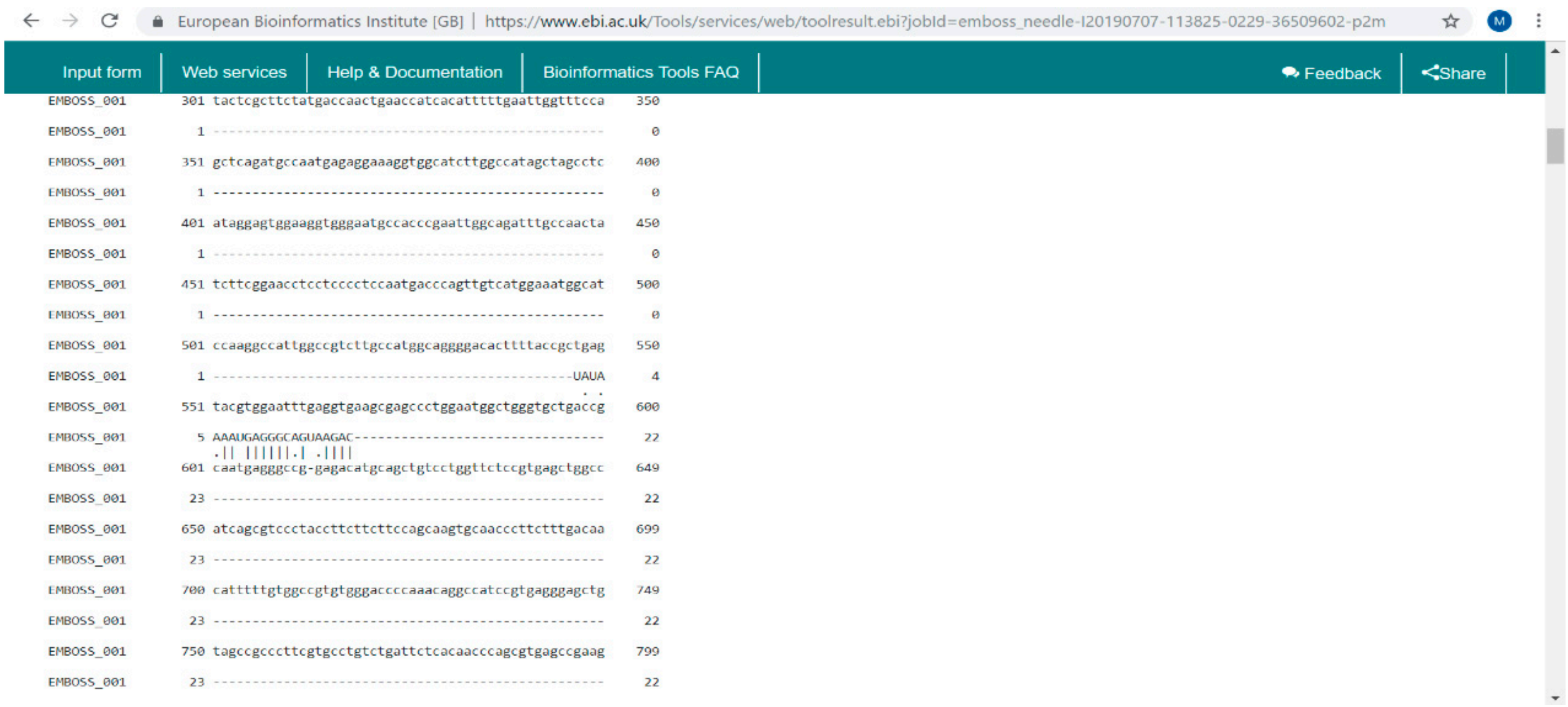

Figure S2(o)**:** Snapshot shows alignment of *miR-1 miRNA* with *mTOR mRNA* ***(available at https://www.ebi.ac.uk)***

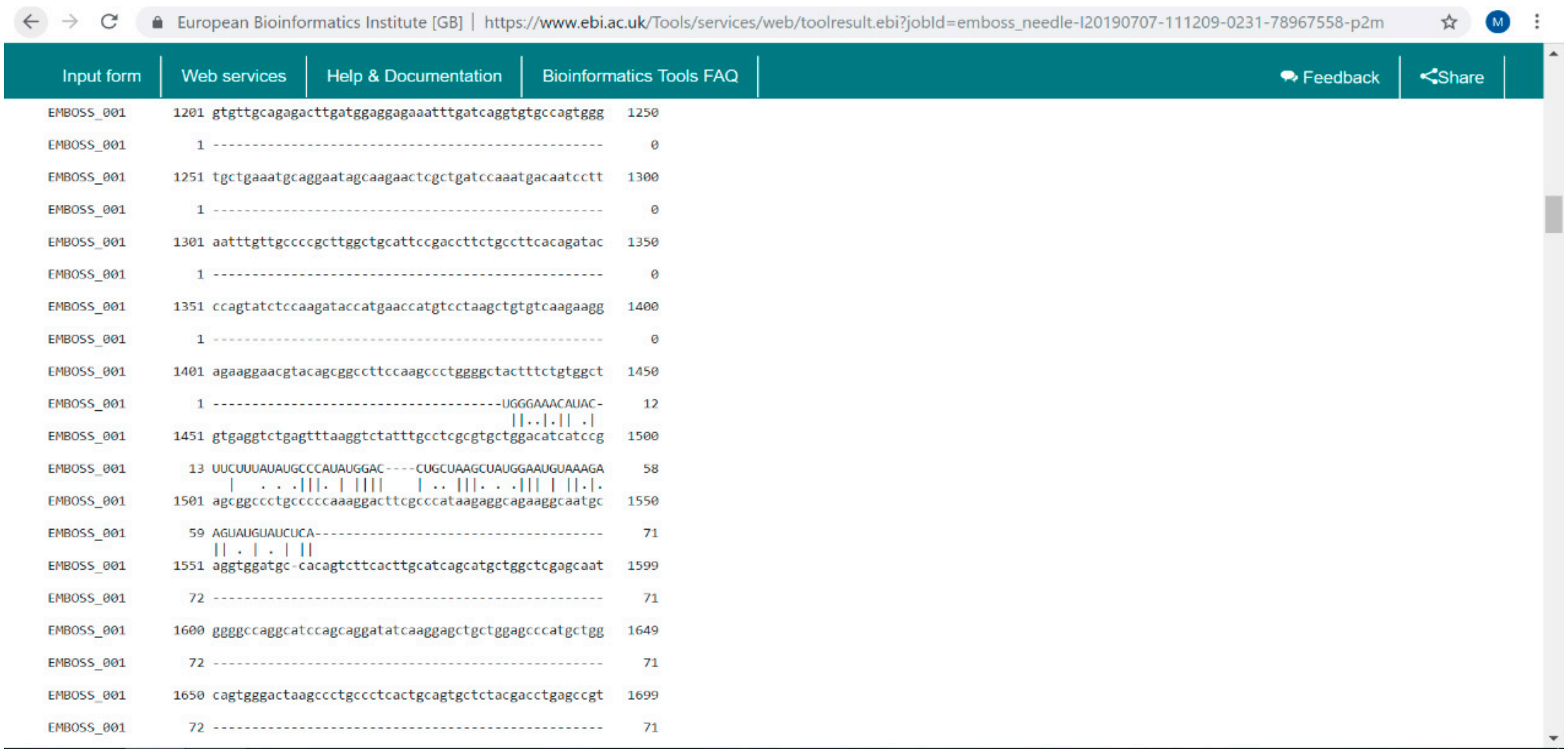


```
EMBOSS_001      1201 gtgttgcagagacttgatggaggagaaatttgatcaggtgtgccagtggg   1250

EMBOSS_001         1 --------------------------------------------------      0

EMBOSS_001      1251 tgctgaaatgcaggaatagcaagaactcgctgatccaaatgacaatcctt   1300

EMBOSS_001         1 --------------------------------------------------      0

EMBOSS_001      1301 aatttgttgccccgcttggctgcattccgaccttctgccttcacagatac   1350

EMBOSS_001         1 --------------------------------------------------      0

EMBOSS_001      1351 ccagtatctccaagataccatgaaccatgtcctaagctgtgtcaagaagg   1400

EMBOSS_001         1 --------------------------------------------------      0

EMBOSS_001      1401 agaaggaacgtacagcggccttccaagccctggggctactttctgtggct   1450

EMBOSS_001         1 -------------------------------------UGGGAAACAUAC-     12
                                                          ||..|.|| .|
EMBOSS_001      1451 gtgaggtctgagtttaaggtctatttgcctcgcgtgctggacatcatccg   1500

EMBOSS_001        13 UUCUUUAUAUGCCCAUAUGGAC----CUGCUAAGCUAUGGAAUGUAAAGA     58
                       |   . . .|||. | ||||    | .. |||. . .||| | ||.|.
EMBOSS_001      1501 agcggccctgcccccaaaggacttcgcccataagaggcagaaggcaatgc   1550

EMBOSS_001        59 AGUAUGUAUCUCA-------------------------------------     71
                     || . | . | ||
EMBOSS_001      1551 aggtggatgc-cacagtcttcacttgcatcagcatgctggctcgagcaat   1599

EMBOSS_001        72 --------------------------------------------------     71

EMBOSS_001      1600 ggggccaggcatccagcaggatatcaaggagctgctggagcccatgctgg   1649

EMBOSS_001        72 --------------------------------------------------     71

EMBOSS_001      1650 cagtgggactaagccctgccctcactgcagtgctctacgacctgagccgt   1699

EMBOSS_001        72 --------------------------------------------------     71
```

figure S3: Snapshot shows alignment between lncRNA and the 2 guides, flanking the sequence of lncRNA and miRNAs interaction (available at https://www.ebi.ac.uk).

1st guide alignment with lncRNA

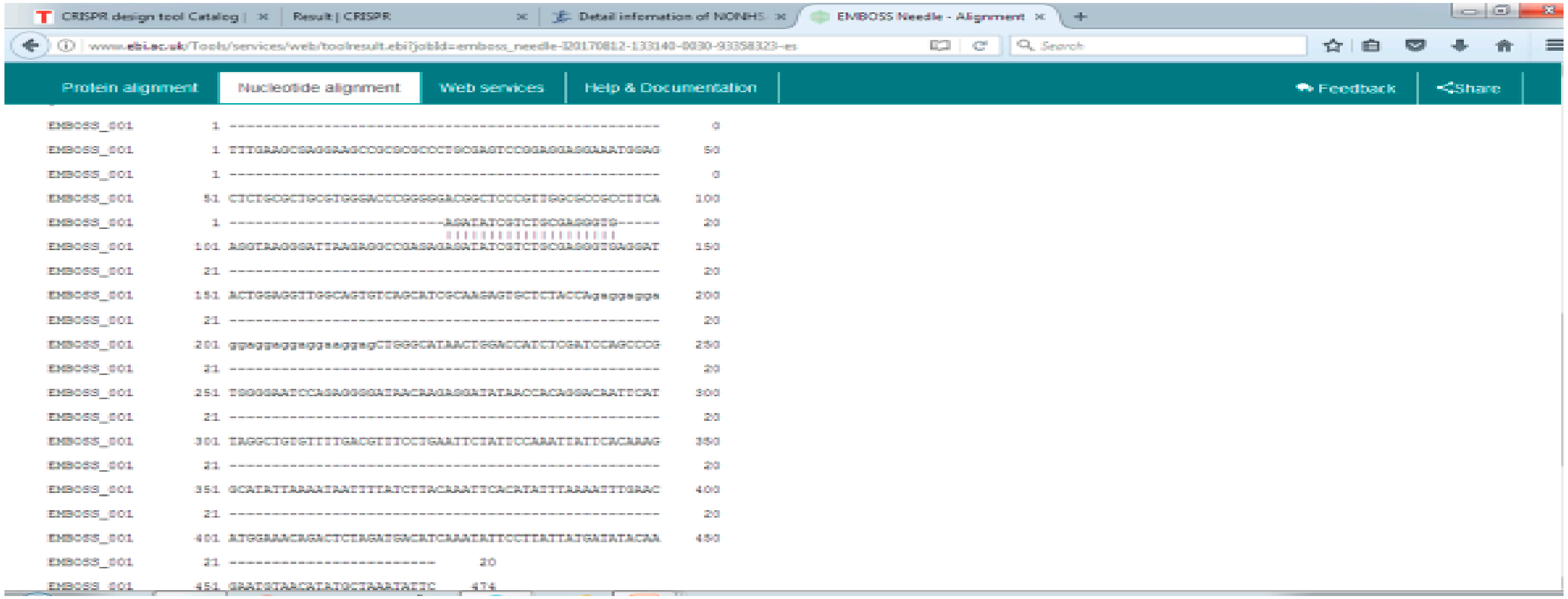

2nd guide alignment with lncRNA

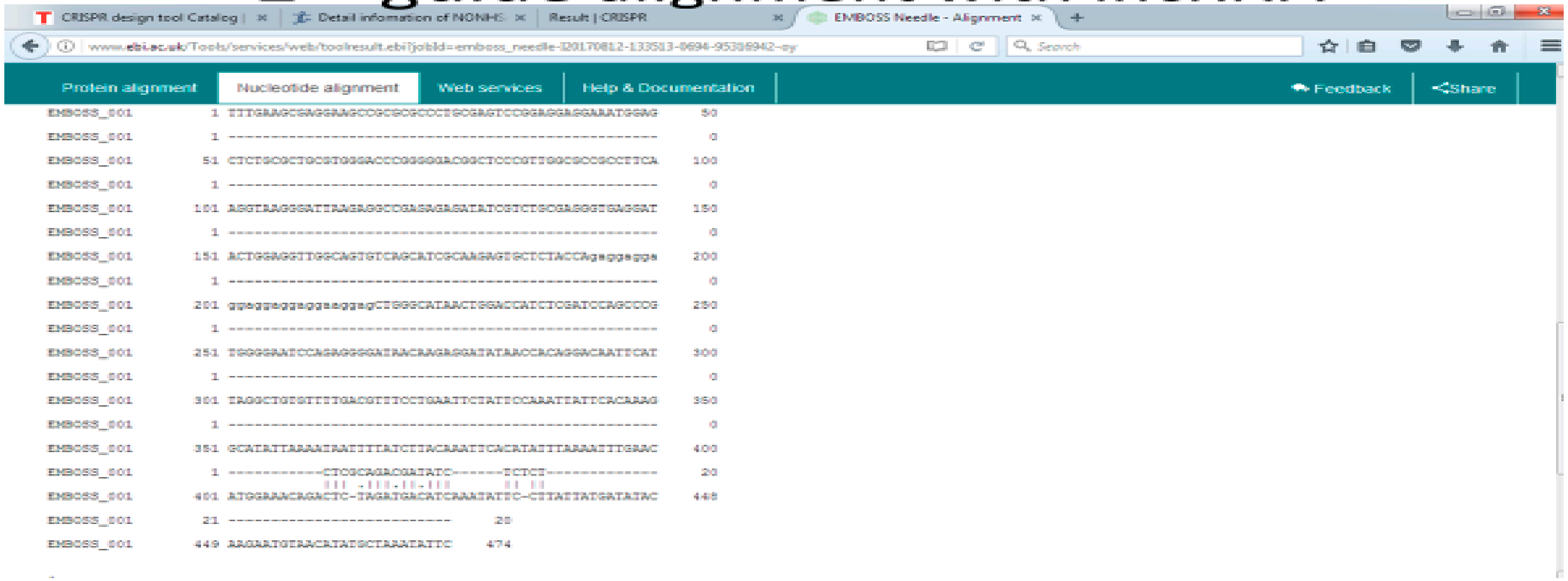

Table S2: Sequences of the forward and reverse primers of the 2 guide RNAs:

**Sequence**	**Primer name**	**GUIDE**
**TAATACGACTCACTATAGAGATATCGTCTG CGAG**	**IVT-NONHSAT0584-gRNA-T1-fwd**	**1st guide**
**TTCTAGCTCTAAAACCACCCTCGCAGACGA TATC**	**IVT-NONHSAT0584-gRNA-T1-rev**	
**TAATACGACTCACTATAGCTCGCAGACGAT ATCT**	**IVT-NONHSAT0584-gRNA-T5-fwd**	**2nd guide**
**TTCTAGCTCTAAAACAGAGAGATATCGTCT GCGA**	**IVT-NONHSAT0584-gRNA-T5-rev**	
